# Supplementary figures and images for: Identification of naturally processed Zika virus peptides by mass spectrometry and validation of memory T cell recall responses in Zika convalescent subjects
Source: PLoS One. 2021 Jun 2;16(6):e0252198. doi: 10.1371/journal.pone.0252198 (PMC8171893; doi:10.1371/journal.pone.0252198)

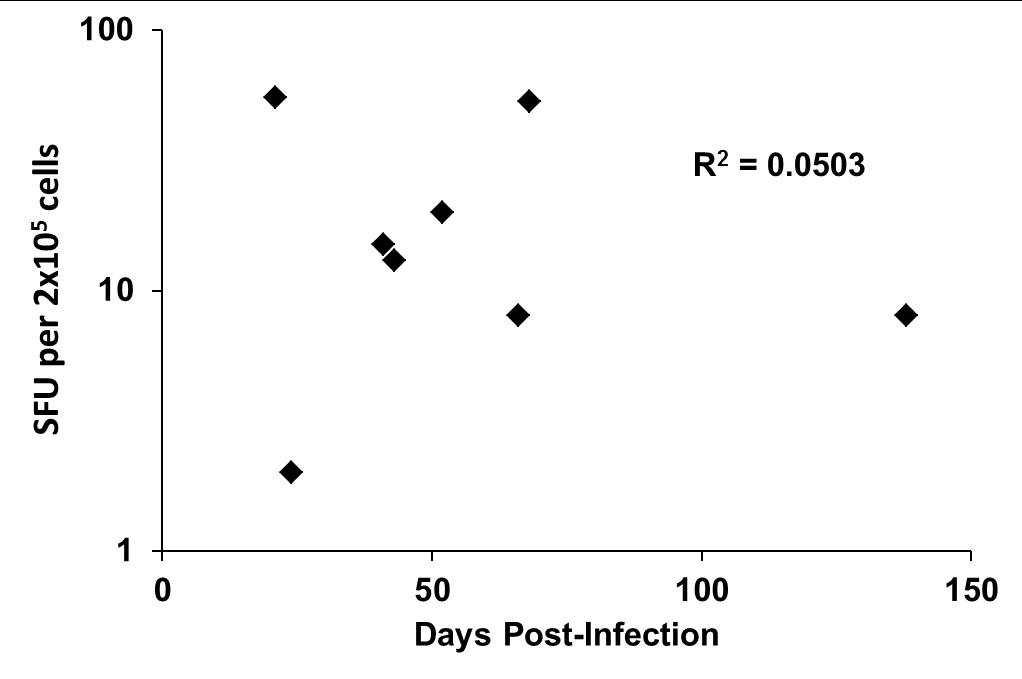

Supplement: S1 Fig — (JPG) [file pone.0252198.s001.jpg]

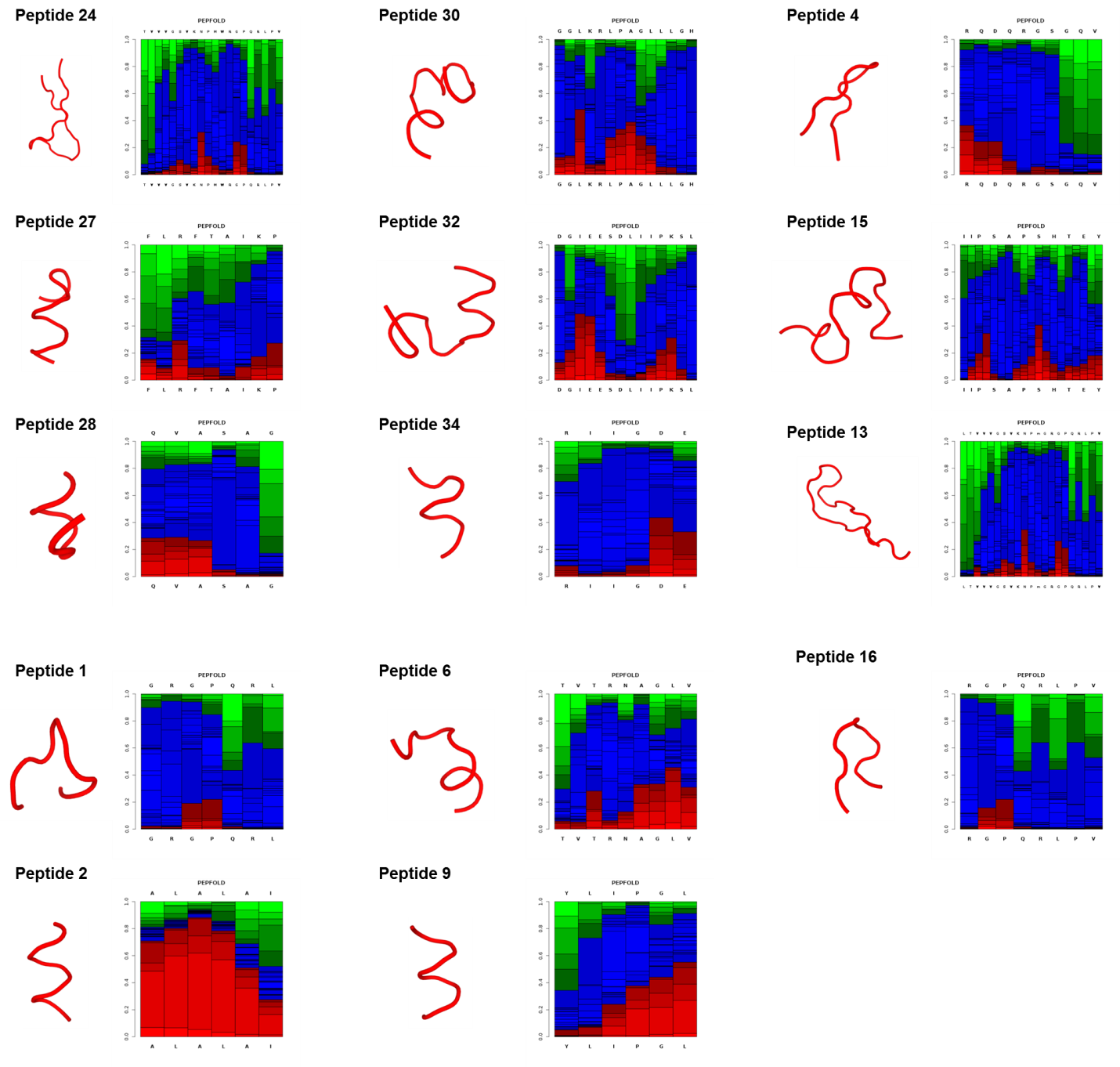

Supplement: S2 Fig — Best fit models for all peptides in the candidate and comparator subsets are shown alongside the probabilities of local structural properties by amino acid position. Red = helical, blue = coiled, green = extended. (PNG) [file pone.0252198.s002.png]

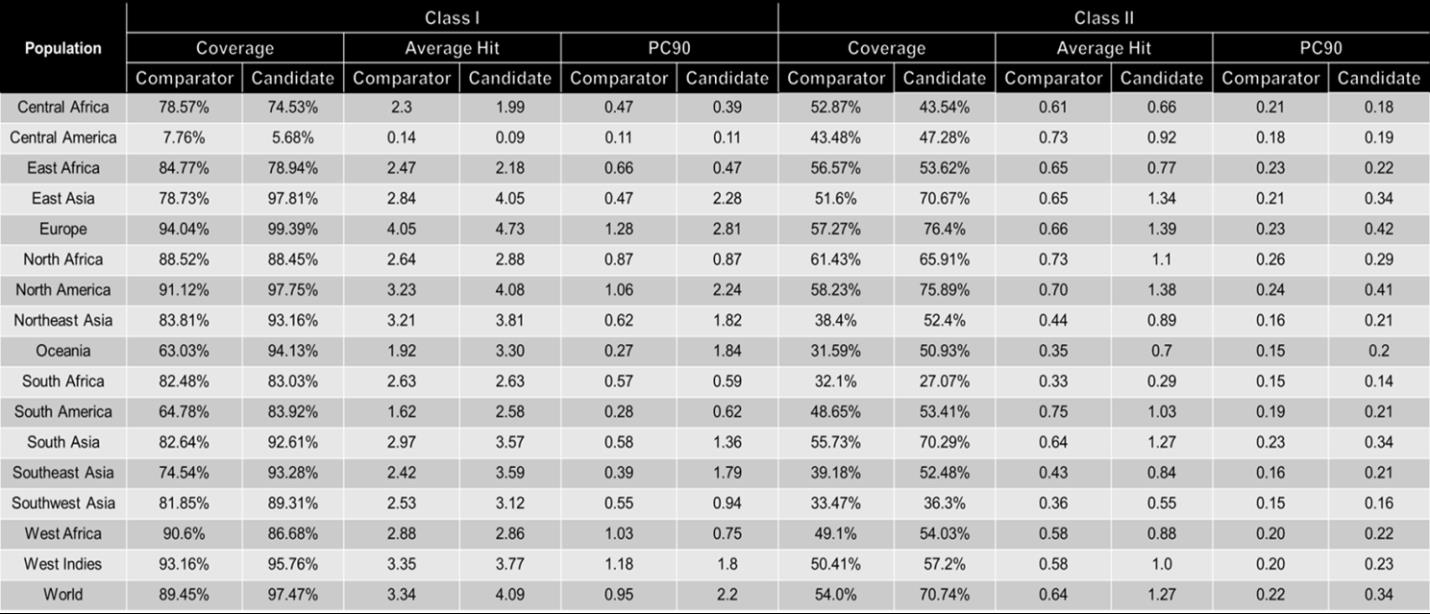

Supplement: S1 Table — (JPG) [file pone.0252198.s003.jpg]

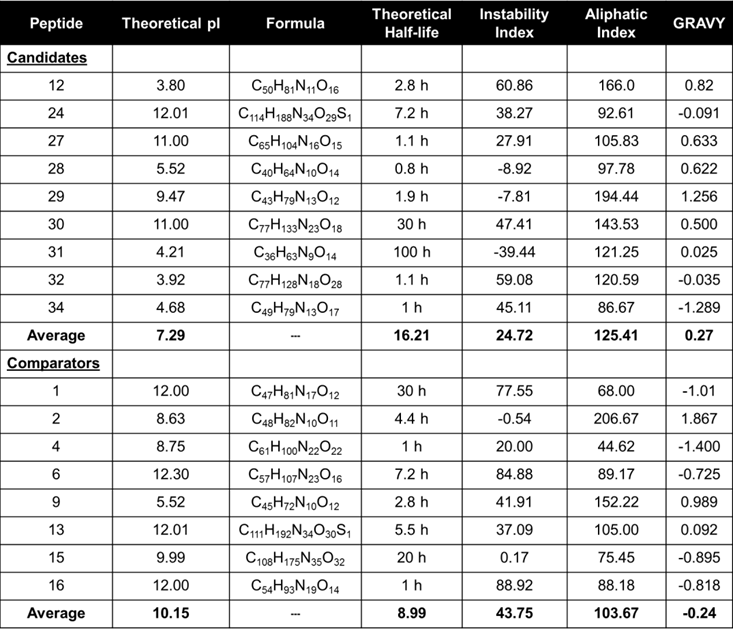

Supplement: S2 Table — Theoretical pI represents the calculated isoelectric point for the peptide. The half-life was calculated based on models of cellular processes in cultured mammalian reticulocytes. The aliphatic index is the relative volume occupied by aliphatic amino acid side chains. GRAVY is the sum of hydropathy values for each amino acid in the peptide sequence. (PNG) [file pone.0252198.s004.png]
