# Supplementary material for: Identification of naturally processed Zika virus peptides by mass spectrometry and validation of memory T cell recall responses in Zika convalescent subjects
Source: PLoS One. 2021 Jun 2;16(6):e0252198. doi: 10.1371/journal.pone.0252198 (PMC8171893; doi:10.1371/journal.pone.0252198)
Supplement: Attachment — Submitted filename: Response to reviewers.doc [file pone.0252198.s005.doc]

“Identification of naturally processed Zika virus peptides by mass spectrometry and validation of memory T cell recall responses in Zika convalescent subjects”

PONE-D-21-03126

March 24, 2021

Response to Reviewers’ comments

Dear Scheherazade Sadegh-Nasseri:

Thank you for these helpful comments from reviewers. We have considered them in depth and responded to each comment or request in detail. We trust these responses will prove acceptable and look forward to your response.

The point-by-point response to reviewers’ comments can be found below.

**Reviewers’ comments:**

**Reviewer #1:**

*Comments to the authors:*

The authors have produced an interesting work trying to identify processed peptides that could activate a cellular immune response against Zika virus. The overall conclusions are significant, and I think contribute to the overall topic of generating a potential vaccine against this disease.

I feel that the following areas require a response and defense from the authors:

1. Identified peptides using a cell lines that are homozygous for HLA-A*02:01 and the HLA-B that this cell expressed (data do not show in the manuscript) only generate peptides presented by these molecules and for instance only produce a proper T-cell response in those individuals that expressed the same alleles of MHC-I molecules

*Authors’ response:* We direct the reviewer to our revised statements in the Methods section regarding the other HLA alleles for the Priess cell line. For the HLA-B in question, Priess cells are documented to express HLA-B*15. While we would certainly predict a strong T cell response in individuals sharing the same alleles as the Priess cell line, some of the peptides identified by our experimental approach are likely to bind and be presented by more than one HLA molecule (e.g., other alleles within an HLA supertype)—thus stimulating responses in a range of individuals and not just those with HLA types that are matched to Priess cells.

2. Although those peptides could be presented by another MHC-I molecules as you explain in the manuscript, what is the affinity of those peptides to other MHC-I molecules

*Authors’ response:* We refer the reviewer to Table 2 in our manuscript for the detailed list of HLA class I molecules that peptides are predicted to bind based on our approach. While the predicted affinities range widely for each peptide-HLA interaction, most peptides were predicted to bind with low nanomolar to low micromolar affinities, which is reflected by the percentile cutoffs used in our Methods section. We note that we selected our predicted peptides with a conservative percentile rank threshold, which selected for the best binders (e.g., those with the highest affinity). However, we should also note that predictive algorithms are not a perfect representation of actual biology, and the true binding affinities of the peptides listed here may differ *in vivo*.

3. Could you explain how those peptides generated for some MHC-I molecules could immunize people that do not present the MHC-I molecules that could bind those potential peptides?

*Authors’ response:* We disagree that peptides presented by one HLA allele cannot bind to and be presented by other HLA alleles. Peptide promiscuity in HLA presentation clearly happens and is the entire premise behind HLA supertypes. Please note that while the peptides identified by mass spectrometry in our study would have only been isolated from the HLA alleles expressed by the Priess cells, our immunoinformatic analysis identified numerous other HLA alleles that these peptides are predicted to bind with relatively high affinities. We used these predicted alleles for our calculations of population coverage based on known allele frequencies. From this, we determined that the candidate peptides listed in Table 2 provided >97% global coverage for HLA class I alleles and >70% global coverage for HLA class II alleles. This is supportive evidence that a vaccine formulated with these peptides could be used to immunize a significant proportion of the population against Zika. While such a vaccine may not be as effective in certain individuals (based on their HLA type), the threshold for herd immunity could easily be reached if the vaccine achieved this level of coverage in the population. Further experimentation *in vitro* and *in vivo* are obviously warranted to confirm these predictions.

4. Why do you not genotype the patients whose plasma is used in the T-cell ELISPOT in order to explain how some patients have a strong immune response against the peptides while others do not?

*Authors’ response:* This is an excellent suggestion. We sought to do this but were provided with a very limited quantity of PBMCs for these subjects. We exhausted our supply of PBMCs conducting and verifying the results of the ELISpot experiments, so we did not have any biospecimens remaining to conduct genotyping (see Lines 362-363). This is something that we would like to accomplish in future work to more directly relate the immunological response with a subject’s HLA type.

5. Do you not think that based only on in silico preditions could have limitations in order to clarify the immune response against those peptides?

*Authors’ response:* We certainly agree that *in silico* predictions are not without limitations; however, given our limited accessibility to biospecimens from ZIKV convalescent subjects, we felt that an immunoinformatics analysis was best suited to address whether the peptides identified by our mass spectrometry-based approach may have the potential to elicit a response in individuals with different HLA types. By combining predictive information on peptide binding with estimates of population coverage for different HLA alleles, we feel that the results of our study provide preliminary evidence of peptides that may be of great interest in vaccine development studies due to their putatively broad population reactivity.

**Reviewer #2**

*Comments to the authors:*

The manuscript by Crooke et al. shows the identification by mass spectrometry of HLA ligands derived from Zika virus and the study of memory T cell response in Zika convalescent subjects. The study of T cell epitopes from Zika virus, and other viral pathogens, is of outstanding interest at this moment and are required for the design of future vaccines.

However, I have some concerns regarding some points of the manuscript and the how the study was made:

MAJOR CONCERNS:

1. Acid stripping treatment works very well for HLA-I molecules, but it is not as good for HLA-II molecules (as they are stabilized at low pH). Authors should show that with this protocol, ligands from HLA-II molecules are obtained.

*Authors’ response:* A method for isolation of immunogenic peptides from HLA class I complexes was originally proposed by Storkus in 1993 (see Storkus WJ et al, J of Immunotherapy, 1993). While we acknowledge that acid stripping is more efficient for isolating peptides associated with HLA-I molecules (as the reviewer points out), this approach has been previously shown to be an effective method for stripping peptides from HLA-II molecules (see Ramachandra et al, J Immunol 1999). We emphasize that our primary goal in this work was an exploratory analysis of potential vaccine targets and not to definitively characterize the HLA presentation of the ZIKV peptides identified by our mass-spectrometry approach. Such endeavors will be part of future work where we validate the targets identified here (as well as potential new targets) by using immunoaffinity purification to isolate peptide:HLA complexes.

2. The putative HLA ligands was purified after the treatment of Priess cells, after two washes with PBS, with an acid buffer, with the consequent peptide stripping. The fact that no HLA immunoprecipitation was done and as mass spectrometry is a highly sensitive technique, any viral peptide remaining in the medium would probably be detected. The authors indicate that a total of 2305 MS/MS spectra were analyzed. However, they do not define the criteria to consider a peptide as correct (Mascot score, etc). In addition, the authors do not show the number of peptides (from human, bovine and Zika virus) with a correct assigned sequence. Thus, it is important that authors add a supplementary table with the list containing the sequence of all peptides identified, the parameters of the search (Mascot score, etc) and the assignation as a binder of one HLA molecule expressed by the cells. This is important to see if the peptides contain the anchor motifs to bind to the HLA molecules expressed in Priess cells (HLA-A*02:01, -B*15 and DRB1*04:01).

*Authors’ response:* We believe the level of detail provided in the “MS/MS data analysis” subsection of the Methods sufficiently outlines our workflow for identifying peptides from ZIKV (as well as peptides of human and bovine origin) and correctly assigning their origin. We elected not to include the full list of peptide sequences for two reasons: 1) the list files were incredibly large, and we felt their inclusion did not significantly contribute to the overall findings of our report; and 2) several of the ZIKV peptides not presented in this work are components of active research that will be publicly disclosed in a later publication. We again stress that our primary goal was to identify preliminary targets for a peptide-based vaccine, and we should note that any given peptide sequence may bind to more than one HLA molecule (as noted in the predictions from our immunoinformatic analysis). While we concede that the reviewer is correct in noting that remnant viral peptides in the media that were not HLA associated may have been identified, we note two things: 1) we used size-exclusion filtration to isolate peptides from remnant HLA molecules and other proteins in the wash buffer—this step would have also presumably removed any remaining source of viral peptides (e.g., viral particles, viral proteins) that were not already digested and processed; and 2) the list of peptides that we carried forward from our mass-spectrometry analysis were those with the highest confidence identified in two biological replicate experiments. We have added a statement in the Methods to make this clearer. Finally, the ability of these peptides to elicit T cell recall responses in infected subjects demonstrates that those peptides we identified as promising candidates are indeed capable of being recognized by HLA molecules.

3. The HLA binding prediction was made with NetMHCpan 4.0 for HLA-I and NetMHCIIpan 3.2 for HLA-II. This is software widely used for this goal. However, the authors say that "The complet list of predicted peptides was filtered for sequences matching (or nested within) selected peptide sequences..." both for HLA-I and HLA-II. I consider that it is correct for HLA-II, but not for HLA-I, as the peptide ends are fixed and interacting with the HLA molecule. I would like to know the explanation to this sentence as maybe I have not understood how the filter was applied.

*Authors’ response:* We apologize for the confusion and have amended our statement to be clearer on the purpose of filtering the sequences. The predictions we ran identified peptides across the entire sequence of the ZIKV protein(s). Our intent here was to down-select from the computational predictions to identify consensus sequences that were matched to peptides that we identified by mass-spectrometry analysis. These consensus sequences could have been either an exact match to a peptide we identified or partially/fully contained within a peptide that we identified. Because exact matches between the predicted and mass-spectrometry-identified peptides were not present for all peptides, we used the consensus sequence of the predicted peptide to inform our decision for selecting peptides identified by mass spectrometry that had partial sequence homology. Our approach was such that using peptides identified from *in vitro* experiments (i.e., the peptides identified by mass spectrometry) was of higher priority than using peptides identified from the binding predictions, as any computational method may not completely replicate true biological processes and therefore may not identify all peptides that can be identified experimentally.

4. I do not understand the Table 2. For example, peptide 24 is clearly too long to bind with enough affinity to any HLA class I molecule. Thus, I consider that this peptide should not be considered as a putative binder for HLA-I molecules. The predicted binding affinity should be shown (the "Affinity (nM)" value is a good parameter to indicate the affinity of a peptide for a specific HLA molecule).

*Authors’ response:* We agree with the reviewer that the length of certain peptides identified by our mass-spectrometry analysis would preclude them from binding certain HLA molecules. While the prediction algorithms identified multiple peptides containing the same or similar consensus sequences, we did not identify all variations of these peptides by mass spectrometry, and we certainly did not have the available samples to synthesize and test all predicted sequences. Therefore, the larger sequences shown in Table 2 (presumably HLA-II binders) are peptides that were identified by mass spectrometry, and reactivity with HLA-I alleles was noted as smaller sequences nested within this parent sequence were predicted to bind HLA-I molecules. Because we do not know how these peptides would be processed upon delivery in a vaccine formulation, we opted to err on the side of caution and present HLA alleles from both class I and class II that were predicted to bind any part of these peptides. We have added a footnote to Table 2 for clarification.

We feel that the scoring threshold applied was a much more comprehensive metric to evaluate peptide binding as it incorporated binding affinity and several other metrics (as detailed on the webpages for each of the algorithms used). For clarification, all peptides discussed for detailed analysis in this report had binding affinities in the low nanomolar to low micromolar range for the various alleles.

5. The manuscript lacks supplementary figure 2 and table 2. At least, I could not see them.

*Authors’ response:* We apologize for this omission. Figure S2 and Table S2 are uploaded with the revised version of the paper.

MINOR CONCERNS:

1. The complete HLA typing of Priess cells should be shown in the manuscript, as other HLA molecules than A*02:01 or DRB1*04:01 can present peptides on the cell surface.

*Authors’ response:* We have added a descriptive statement in the Methods that details the complete HLA typing of the Priess cell line (as documented by Millipore Sigma and the ECACC).

**End of response to reviewers’ comments.**

We trust these responses will prove satisfactory upon review and look forward to having the manuscript accepted. We thank the reviewers for their careful and insightful comments. We believe the manuscript is stronger for them.

Sincerely,

Gregory A. Poland, M.D.
