# Supplementary material for: Identification of naturally processed Zika virus peptides by mass spectrometry and validation of memory T cell recall responses in Zika convalescent subjects
Source: PLoS One. 2021 Jun 2;16(6):e0252198. doi: 10.1371/journal.pone.0252198 (PMC8171893; doi:10.1371/journal.pone.0252198)
Supplement: Attachment — Submitted filename: Response to reviewers 2021-05-06.doc [file pone.0252198.s006.doc]

“Identification of naturally processed Zika virus peptides by mass spectrometry and validation of memory T cell recall responses in Zika convalescent subjects”

PONE-D-21-03126

May 7, 2021

Response to Reviewers’ comments

Dear Scheherazade Sadegh-Nasseri:

Thank you for these helpful comments from reviewers. We have considered them in depth and responded to each comment or request in detail. We trust these responses will prove acceptable and look forward to your response.

The point-by-point response to reviewers’ comments can be found below.

**Reviewers’ comments:**

**Reviewer #1:**

*Comments to the authors:*

Stephen N Crooke and co-authors have addressed all my concerns adequately. I recommended publication.

*Authors’ response:* We thank the reviewer for the critique of our manuscript and are glad to have addressed their concerns.

**Reviewer #2**

*Comments to the authors:*

I want to thank the responses to my previous comments. The authors have cleared most of my doubts out.

My unique request to the authors to consider the manuscript acceptable to publication is to add to the manuscript a sentence similar to that included in their comment to my first question: "...our primary goal in this work was an exploratory analysis of potential vaccine targets and not to definitively characterize the HLA presentation of the ZIKV peptides identified by our mass-spectrometry approach. Such endeavors will be part of future work where we validate the targets identified here (as well as potential new targets) by using immunoaffinity purification to isolate peptide:HLA complexes".
I consider this is important as, in my opinion, the authors have not completely demonstrated in this work that the peptides are bona fide HLA ligands.

*Authors’ response:* We agree that our work does not prove HLA binding and presentation for the peptides identified in the manuscript. Therefore, we have added the text suggested by Reviewer #2 in the Discussion (Line 382) and have removed the following text: “we focused on peptides that were naturally processed and presented on HLA molecules following viral infection.”

**End of response to reviewers’ comments.**

We trust these responses will prove satisfactory upon review and look forward to having the revised manuscript accepted. We thank the reviewers for their careful and insightful comments. We believe the manuscript is stronger for them.

Sincerely,

Gregory A. Poland, M.D.
